# The Role of the Oculomotor System in Updating Visual-Spatial Working Memory across Saccades

**DOI:** 10.1371/journal.pone.0161829

**Published:** 2016-09-15

**Authors:** Paul J. Boon, Artem V. Belopolsky, Jan Theeuwes

**Affiliations:** Department of Experimental and Applied Psychology, Vrije Universiteit, Amsterdam, The Netherlands; The University of Melbourne, AUSTRALIA

## Abstract

Visual-spatial working memory (VSWM) helps us to maintain and manipulate visual information in the absence of sensory input. It has been proposed that VSWM is an emergent property of the oculomotor system. In the present study we investigated the role of the oculomotor system in updating of spatial working memory representations across saccades. Participants had to maintain a location in memory while making a saccade to a different location. During the saccade the target was displaced, which went unnoticed by the participants. After executing the saccade, participants had to indicate the memorized location. If memory updating fully relies on cancellation driven by extraretinal oculomotor signals, the displacement should have no effect on the perceived location of the memorized stimulus. However, if postsaccadic retinal information about the location of the saccade target is used, the perceived location will be shifted according to the target displacement. As it has been suggested that maintenance of accurate spatial representations across saccades is especially important for action control, we used different ways of reporting the location held in memory; a match-to-sample task, a mouse click or by making another saccade. The results showed a small systematic target displacement bias in all response modalities. Parametric manipulation of the distance between the to-be-memorized stimulus and saccade target revealed that target displacement bias increased over time and changed its spatial profile from being initially centered on locations around the saccade target to becoming spatially global. Taken together results suggest that we neither rely exclusively on extraretinal nor on retinal information in updating working memory representations across saccades. The relative contribution of retinal signals is not fixed but depends on both the time available to integrate these signals as well as the distance between the saccade target and the remembered location.

## Introduction

Visual-spatial working memory (VSWM) helps us maintain and manipulate visual information in the absence of sensory input. While traditionally regarded as a separate, higher order cognitive function, VSWM it is more likely to emerge from already existing lower level systems, arising through the coordinated recruitment of sensory and motor processing systems [1,2]. One influential idea is that the maintenance of spatial information involves the same neural circuits used to plan and execute eye movements [3]. In accordance with this idea, it was shown that remembering the location of an object generates corresponding activity within the oculomotor system. This activity competes with saccade goals, making the eyes curve around remembered locations [4–7]. Further evidence for a functional relationship between working memory and the eye movement system comes from studies in which the ability to plan eye movements to certain locations was experimentally impaired by abducting the eye in the orbit. This resulted in deficits in the maintenance of information at these specific locations [8,9]. In addition, neurophysiological recordings in non-human primates show that the locations of memorized targets are represented in regions known to be involved in attention and the preparation of eye movements, such as the lateral intraparietal area (LIP; [10] and frontal eye fields (FEF; [11,12]. In humans, memorized information appears to be represented in homologous areas [13,14].

Given that the oculomotor system is retinotopically organized, the locations of memorized stimuli need to be updated with each saccade; every time the eyes move, the visual environment sweeps across the retina. As a consequence, all relevant locations will be represented by a different group of retinotopically tuned neurons after the movement is completed. In the present study we investigated the role of the oculomotor system in keeping track of the locations of remembered objects across saccades. We tried to probe to what degree we rely on extra-retinal oculomotor signals and postsaccadic retinal information in maintaining spatial working memory across saccades.

The question of how visual information is maintained across saccades has preoccupied the minds of researchers for several centuries. One group of theories, dating back to Helmholtz [15,16] emphasized the role of so-called extraretinal signals. Specifically, they argued that when an eye movement is launched, a copy of this motor program (i.e. the efferent copy) is retained, which is later used for correction or cancellation of the displacement of retinal image induced by the eye movement (i.e. the cancellation theories). Consistent with this notion is the finding that shortly before an eye movement cells begin to respond to stimuli that will lie in their classic receptive field after the saccade has been executed. This “predictive remapping” was first discovered in area LIP of rhesus macaques, and has subsequently also been observed in other brain areas involved in oculomotor control, such as FEF ([12,17] and the superior colliculus (SC; [18,19]. Using fMRI, similar predictive remapping effects were found in the posterior parietal cortex of humans [20].

Although remapping has long been interpreted as transient shifts of receptive fields, this view has recently been questioned. Instead, pre-activating of cells in anticipation of postsaccadic input may be a more appropriate mechanism underlying remapping [21]. This activity can serve as a pointer to the locations of relevant objects, enabling us to keep track of them across saccades. This is view congruent with the fact that neurons with remapping properties have mainly been found in LIP, FEF, and SC; all structures that are thought to contain salience maps for spatial attention and saccade targets [22–24]. Furthermore, several studies showed that before a saccade is executed, attention shifts to the retinotopic location that a relevant stimulus will occupy after the saccade [25–28]. If the function of remapping is to keep track of the locations of relevant objects across saccades, it is not unlikely that the same mechanism also underlies the updating of memorized locations.

Even though there is quite some empirical evidence for the existence of a cancellation mechanism driven by the extraretinal signals, cancellation theories cannot explain the finding that observers tend to miss target displacements if they occur during a saccade (e.g., [29–33]. Remarkably, saccade target displacements up to one third of saccade amplitude often go unnoticed [29]. The information about the target location is not lost, because when the target is not continuously present before and after the saccade but reappears at a displaced location after a short blank interval after the saccade (SOA of 50 ms), the displacements are detected with a remarkably high accuracy [31,34,35]. This “target blanking” shows that prediction about the target location based on the extraretinal signal is highly accurate, but that this information is ordinarily discarded in normal perception. Instead, the visual system relies on the null hypothesis that the visual world is stable across saccades (i.e. the assumption theories). As long as there is a rough match between pre- and postsaccadic stimulus location and identity, the visual world is realigned with these objects even if large intrasaccadic displacements occur. In line with this, several studies demonstrated that an object’s position is encoded according to its relative spatial relationship to other stimuli in the environment. The detection of object displacement relies to a large extent on changes in position relative to other objects [36–38], and if one object is displaced during a saccade, a second, stationary but briefly blanked object will be perceived as moving into the opposite direction [36,37,39,40]. In this case, the displaced object will serve as a landmark for the blanked object, and the change in spatial relational information is always attributed to the object that was not continuously present before and after the saccade.

In the present study we were interested in the mechanism for updating the location of a remembered stimulus. Note that in typical studies on visual stability there is always a correspondence between the objects visible before and after the eye movement. However, when memorizing a location there is no stimulus present either before or after the saccade. This lack of object correspondence across saccade could lead to different processes of updating spatial information. Because the assumption of stability is never broken updating might rely completely on extra-retinal signals, driving the remapping of neural activity representing the remembered stimulus. Alternatively, the remembered location might be anchored to other objects in the visual scene, such as the saccade target, serving as landmarks for the no longer present stimulus.

To examine this we tried to quantify the relative contribution of extraretinal oculomotor signals and postsaccadic retinal signals in maintaining spatial working memory across saccades. We asked participants to keep a location in memory and then perform a saccade to a different location. During the eye movement the saccade target could be displaced slightly. As described above, such displacements are typically not noticed by participants [29,31]. We assumed that if updating visual-spatial memory relies exclusively on retinal signals, the error in localizing the memorized location would be equal to the target displacement. As the only reference object (the saccade target) is perceived as stable across the saccade, the assumption of stability is not violated, and the ‘assumption theories’ (e.g. [31,41] would predict the displacement to be attributed to imperfect oculomotor coordination (i.e. the oculomotor error). However, if memory relies exclusively on extraretinal signals, then the saccade target displacement should have no effect on localizing the memorized location after a saccade. This manipulation allows us to directly assess the extent to which the eye movement system is involved in maintenance of stable visual memory representations.

It has been proposed that maintenance of accurate spatial representations across saccades is especially important for actions, but is less critical for perception [42]. The ability to keep track of a continuously present object does not necessarily require a precise prediction of its post-saccadic location. However, in order to act upon objects and to avoid obstacles, the updating of spatial movement goals is crucial. The fact that neurons with remapping properties are all located in regions involved in motor control seems in line with this idea [10,17,19]. Furthermore, when making a sequence of two eye movements, disruption of the extraretinal signal leads to an inability to make accurate secondary saccades [43]. Strong evidence in favor of an action-perception distinction was provided in a study in which participants were asked to simultaneously make an eye movement and a pointing movement to a visual target. Critically, participants were unable to see their own hand movement. When this target was displaced during the eye movement participants did not notice this, but nonetheless manual pointing movements were immediately corrected to compensate for the change in location [44]. Although the sensory system seems to be unaware of the discrepancy between the expected and perceived location, the motor system seems to have direct access to this information. To take this proposed dichotomy between perception and motor control into consideration we have compared different ways of reporting the location held in memory across saccade. In Experiment 1 participants reported the memorized location using a match-to-sample task, in Experiment 2 they indicated the memorized location by a mouse click and in Experiment 3 they were asked to make another saccade to the location held in memory. If maintenance of locations in memory across saccades is more accurate for action-related tasks, we expected that target displacement would have the strongest influence on localization responses in Experiment 1, the weakest influence in Experiment 3, and a moderate influence in Experiment 2. In Experiment 4 we investigated the spatial profile of the target displacement bias by parametrically varying the distance between the memory cue and the saccade target.

## Experiment 1

In this experiment participants had to remember the location of a stimulus briefly shown in the periphery. During a retention interval an eye movement had to be made. During this eye movement the saccade target could be displaced. Participants had to indicate whether a probe appeared left or right of the remembered location. If memory updating fully relies on cancellation driven by extraretinal oculomotor signals, the displacement should have no effect on the perceived location of the memorized stimulus. However, if postsaccadic retinal information about the location of the saccade target is taken into account, the perceived location will be shifted in the direction corresponding with target displacement.

### Methods

Twelve participants, aged between 20 and 27 (mean 22, all female) received either money or study credit to participate in two 60 minute long experimental sessions, completing on average 922 trials. The present and all following experiments, including the consent procedure, were approved by the local ethics committee of the VU University Amsterdam. Participants received information about the study and their rights and gave a written informed consent. Participants were naïve with respect to the aim of the study and had normal or corrected to normal visual acuity. After completion of the experiment all participants were asked by the experimenter whether they noticed any of the stimuli being displaced during any of the experimental trials, and if so, estimate on how many trials they noticed such a displacement. One participant was excluded because she reported “seeing something jump” on approximately four trials. None of the other participants noticed the displacements.

The experiment was conducted in a darkened room. Stimuli were presented on a 21 inch monitor (monitor type: Samsung 2233RZ) with a spatial resolution of 1680 x 1050 pixels and a refresh rate of 120 Hz. Participants viewed the screen binocularly from a distance of 70 cm, and eye movements were recorded with the Eyelink 1000 (SR Research), sampling at 1000 Hz. The fixation dot and saccade target were an open red circle with a radius of 0.19° and a luminance of 27 cd/m^2^. The memory cue was a blue circle with a radius of 0.23° and a luminance of 9 cd/m^2^.

**Fig 1:**
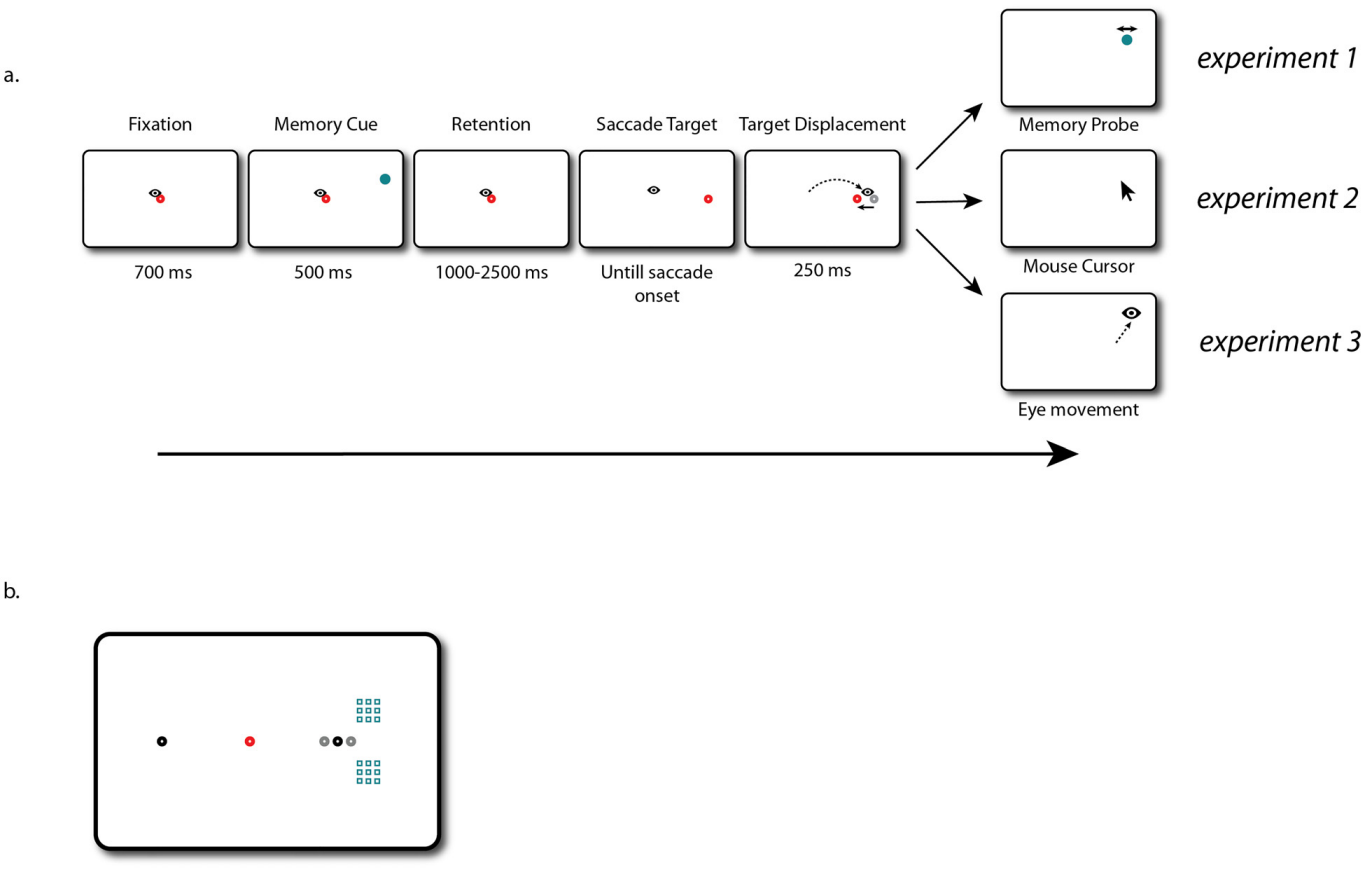
Experimental paradigm of Experiments 1, 2, and 3. a. Participants fixated on the red dot and remembered the location of the blue memory cue. After a variable retention interval the dot jumped either to the right or left, signaling that a saccade had to be executed. Once a saccade was detected, the red saccade target could move backward, forward, or remain in place. After 250 ms saccade target was removed, signaling that a response had to be made. In Experiment 1 a probe appeared. Participants had to indicate with a key press if this probe was located to the left or to the right of the memorized location. In Experiment 2 a mouse cursor appeared and participants had to click on the memorized location. In Experiment 3 a localization eye movement had to be executed to the remembered location. b. Overview of all the possible memory cue locations (blue) in the right hemifield. The red fixation dot indicates the fixation location. The black dots indicate the possible saccade target location, while the gray dots indicate the location of both a backward and forward displaced saccade target.

Participants had to fixate on the red fixation dot randomly placed at a location on the horizontal meridian between 4° left and 4° right from the center of the screen. A blue circle was flashed for 500 ms at one of nine possible locations in each hemifield; at 12.5°, 13.5° or 14.5° of horizontal distance and 2.5°, 3.5°, or 4.5° of vertical distance away from fixation (Fig 1). These peripheral locations were chosen to prevent configurational coding of the memory cue relative to the fixation target. Participants were instructed to keep the location in memory. After a retention interval between 1000 and 2500 ms the fixation dot moved 10° either to the left or to the right, signaling that a saccade had to be made to this location. For online saccade detection a boundary of 3° around fixation location was used. As soon as the recorded eye position exceeded this boundary in the direction of the saccade target (angle within 30° of arc of the target), the gaze contingent changes were introduced. If the eyes moved across the boundary in any other direction than in the direction of the target, no displacement was introduced and the trial was discarded from analysis. If the eyes did move in the right direction, the target was moved either 1.5° in the same direction of the saccade (‘forward’ condition) or 1.5° in the opposite direction (‘backward’ condition), or stayed at the same location (‘no displacement’ condition). All conditions were equally probable. The saccade target remained on the screen for 250 ms after a saccade was detected. After this a probe appeared. This probe was exactly the same as the memory cue and appeared randomly at a location varying between 3.5° in the opposite direction as the saccade and 3° in the same direction as the saccade relative to the original location of the memory cue in steps of 0.5°. Participants responded by pressing the ‘z’ key on the keyboard if they thought that the probe was located to the left of the remembered location, or by pressing the ‘c’ key if they thought the probe was located to the right of the remembered location. To reduce the predictability of saccade direction the memory cue could be located in the same hemifield (2/3 of the trials) or in the opposite hemifield (1/3 of the trials) as the saccade target. However, given the large distance between saccade target and memory cue in the latter situation only trials in which saccade and cue were located in the same hemifield were analyzed.

In order to estimate the amount of displacement leading to chance performance (PNL = perceptual null location), the proportion of forward responses was determined for every probe location and the following two-parameter cumulative Gaussian function was fitted to each individual's data using a least squares optimization method.

$$P\left(Forward\;Response|Probe\;Location\right)=\;\frac{1}{1+e^{-slope\left(Probe\;Location-PNL\right)}}$$

### Results and Discussion

Trials in which a saccade was detected before target onset were discarded. If the saccade was shorter than 7°, had a latency shorter than 80 ms or longer than 600 ms, or did not start at fixation, the trial was discarded. This resulted in loss of 19.7% of the trials. Trials were collapsed across saccade direction (left or right) and cue location (upper or lower hemifield). There was no difference in saccade amplitude between backward, forward, and no displacement conditions (F(2,20) = 1.34 p =.29). A corrective saccade was made in 15% of the trials in the backward and no displacement condition, and 21% of the trials in the forward condition. The probe appeared on average 222 ± 6 ms after landing of the first saccade.

**Fig 2:**
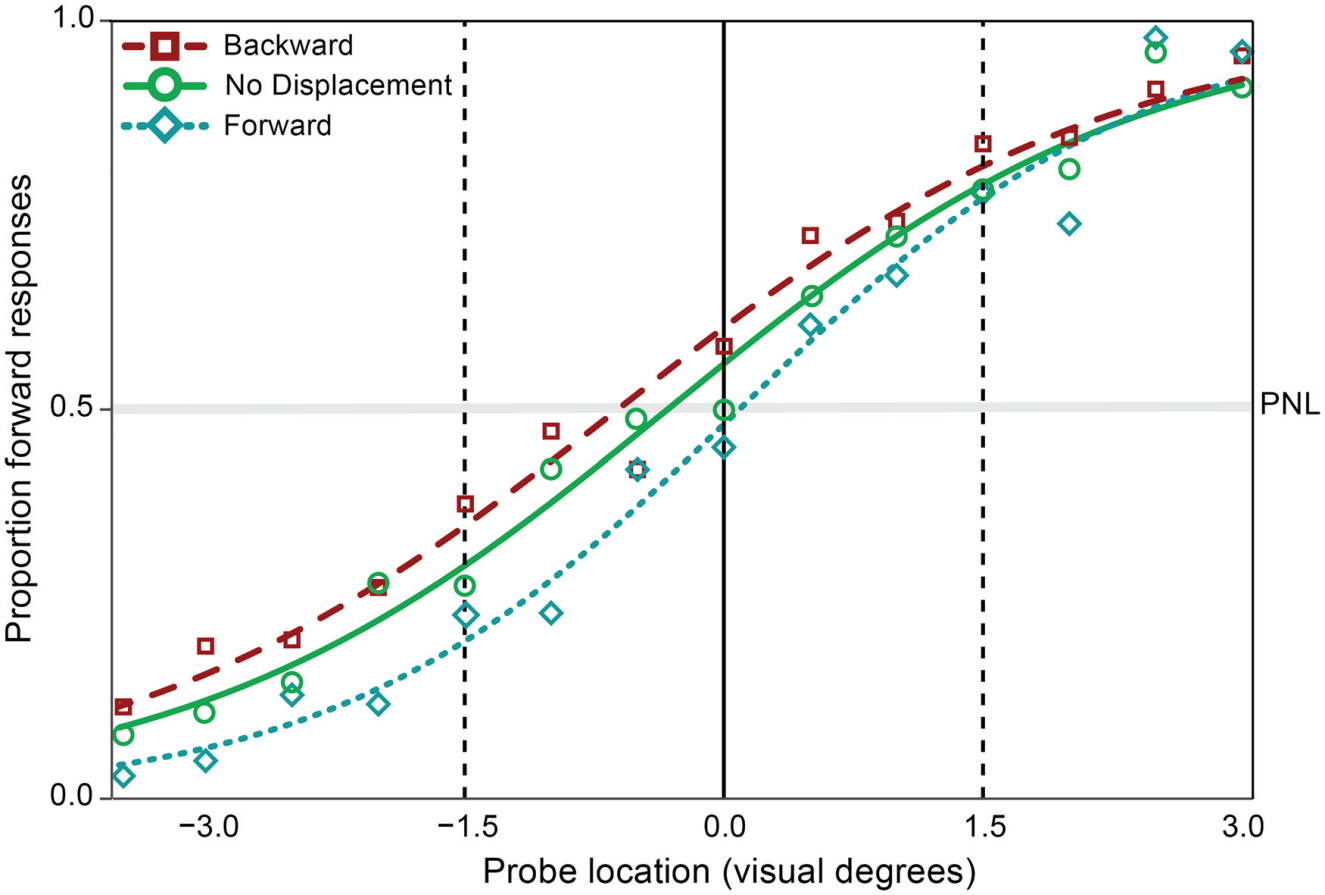
Mean psychometric curves for backward, forward and no displacement conditions in Experiment 1. Probe location is indicated on the x-axis. The dots represent the mean proportion of forward responses in the no displacement (green), backward (red), and forward (blue) condition. The solid vertical line indicates the correct memory location in the three conditions. The dashed vertical lines indicate the displacement size of the saccade target in the ‘backward’ and ‘forward’ condition. The horizontal grey line indicates chance level. The perceptual null location (PNL), the probe location for which there was an equal number of forward and backward responses, is located at the intersection of the psychometric curve with this line.

Mean proportions of forward responses at each probe location for each condition and the average of the fitted psychometric curves for each condition are plotted in Fig 2. In the no displacement condition the average PNL (perceptual null location) was located 0.27° away from the actual location in the opposite direction of the saccade. PNL in the backward and forward conditions was close to the non-displaced location, but target displacement did introduce a small but consistent bias in the direction of target displacement. Averaged across the ‘backward’ and ‘forward’ conditions this bias was 24% of the actual size of target displacement, and a within-participants ANOVA revealed a significant effect of target displacement (backward, forward, or no displacement) on PNL (F(2,20) = 17.09, p<0.001, η_p_^2^ = 0.63). The data were also examined using a Bayes factor ANOVA with default prior scales, comparing the fit of the data under the null hypothesis and the alternative hypothesis. The estimated Bayes factor (alternative/null) suggests that the data are 325 times more likely to occur under the model including the effect of target displacement.

The unnoticed perisaccadic displacement of a target influences the localization of a remembered stimulus. The location at which the probe is perceived at the same location as where the memory cue was presented is shifted in the direction of the displaced target. Although this effect is very consistent across participants, the size of this shift is only a small fraction of the total size of target displacement (24%). Cancellation driven by extraretinal oculomotor signals appears to be the dominant mechanism in determining the location of a remembered stimulus. Nonetheless, directly after saccade landing integration of retinal signals takes place. The question is whether the effect of postsaccadic retinal signals on localization is specific to the visual domain or will also bias actions towards the remembered stimulus.

## Experiment 2

In Experiment 2 participants had to perform a task similar to Experiment 1; the main difference being the response modality that was used to indicate the memorized location. While in Experiment 1 a probe had to be compared to the memorized stimulus, in Experiment 2 a mouse click was used to indicate the memorized location. If memory updating for action relies more heavily on the oculomotor system than memory updating for perception, we expect the memory bias introduced by the retinal signals to be smaller in the mouse pointing task than in the purely perceptual match-to-sample task in Experiment 1.

### Methods

Eight participants, aged between 20 and 27 (mean 23, male: 4) received either money or study credits to participate in a 75 minute experiment, completing 288 trials. They were naïve with respect to the aim of the study and had normal or corrected visual acuity. After completion of the experiment, all participants were asked whether they noticed the target displacement on some trials, and if so, on how many trials they noticed such a displacement. None of the participants noticed any displacements.

The task was very similar to Experiment 1. Participants had to fixate on a red fixation dot and keep a location in memory. After a retention interval a saccade had to be made during which the saccade target could be displaced either 1.5°in the direction of the saccade (‘forward’ condition) or 1.5° in the opposite direction (‘backward’ condition), or stay at the same location (‘no displacement’ condition). The saccade target remained on the screen for 250ms after a saccade was detected. A mouse cursor then appeared at a random location within 2°of horizontal and vertical distance from the screen center and participants clicked on the location where they thought the memory cue had been presented. If the direction of the saccade was more than 30° of arc away from the target, no displacement was introduced and the trial was discarded from the analysis. As before, only the trials in which the cue was presented in the same hemifield as the saccade target (2/3 of the trials) were analyzed.

### Results and Discussion

Trials in which a saccade was detected before the target onset were discarded. If the saccade was shorter than 7°, was faster than 80 ms or slower than 600 ms, or did not start within 2° of fixation the trial was discarded. This resulted in an average loss of 9.9% of all trials. There was no difference in saccade amplitude between backward, forward, and no displacement conditions on the remaining trials (F(2,14) = 0.03, p =.98).

**Fig 3:**
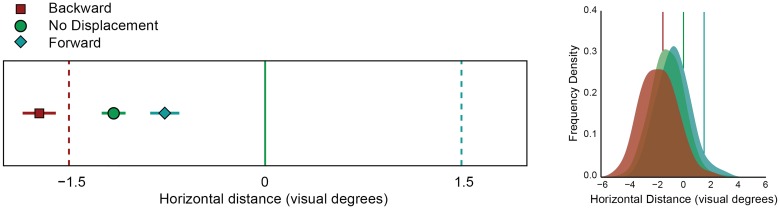
Results of Experiment 2. Left: Mean localization responses in backward, forward and no displacement conditions. The horizontal distance is plotted on the x-axis. The error bars represent 95% within-subject confidence intervals. The solid vertical line indicates the veridical memory location. The dashed vertical lines indicate the displacement size of the saccade target in the backward and forward displacement conditions, respectively. Right: Population density plot of the localization responses relative to veridical location of the memorized stimulus in backward, forward and no displacement. The solid vertical lines indicate the displacement of the saccade target in the backward, no displacement, and forward conditions, respectively.

For each participant the mean clicked positions on the horizontal axis relative to the correct cue location are plotted in Fig 3. There was a general tendency to underestimate the eccentricity of the cue location (-1.14 °), and localization was significantly different between the backward, forward, and no displacement condition (on average 36% of total target displacement, F(2,14) = 63.70, p<0.001, η_p_^2^ = 0.90). There was no difference in localization in the vertical direction between these conditions (F(2,14) = 1.22, p =.32). The data were also examined by estimating a Bayes factor, comparing the fit of the data under the null hypothesis and the alternative hypothesis. The estimated Bayes factor (alternative/null) suggests that the data are 45688 times more likely to occur under the model including the effect of target displacement.

As in Experiment 1, displacement of the saccade target resulted in responses being biased in the direction of the displacement. Interestingly, making a manual mouse pointing movement toward the memorized location resulted in a larger target displacement bias than in the visual comparison task of Experiment 1; the size of this effect was roughly one third of the magnitude of displacement (36%, averaged across forward and backward displacement conditions). This is not in accordance with the hypothesis that the relative contribution of extraretinal oculomotor signals should be larger in a motor task. However, whereas the visual probe in Experiment 1 was presented shortly after saccade landing, mouse pointing responses were rather slow, allowing participants much more time to integrate retinal information before they make an eventual judgment about the memorized location. In addition, when moving a mouse cursor towards a memorized location one is not directly acting upon this location but rather on the mouse, which in turn induces the movement of the cursor. This indirect motor response, in combination with the long response latencies might have led to the relatively large influence of retinal signals on memory updating.

Although all experiments were conducted in a darkened room, the LCD monitor always emits a dim background illumination. This could have functioned as a stable reference frame, perhaps decreasing the effect of target displacement. To test the viability of this notion, we included a control experiment in which a bright white reference frame with edges close to the borders of the monitor was displaced together with the saccade target (see S1 File). This did not increase the target displacement effect, demonstrating that the frame was not used in the post-saccadic calibration of the remembered location.

## Experiment 3

In both Experiment 1 and 2 a small but systematic target displacement bias was found. In Experiment 3 participants were instructed to indicate the remembered location by making a saccade towards it. If working memory is grounded in the oculomotor system, making an eye movement towards the remembered location may be the most direct assessment of memory. This way a representation of the memorized location does not have to be translated to another response modality before a location can be indicated. If the main function of extraretinal signals is to retain action goals across eye movements, as proposed by Bays and Husain [42], there should be little interference of retinal information on these localization saccades.

### Methods

Eight participants aged between 21 and 31 (mean 27, Male: 4) completed 396 trials in a task that was very similar to Experiments 1 and 2. However, localization of the remembered stimulus was achieved by making an eye movement to its location.

### Results and Discussion

Trials in which a saccade was detected before target onset were discarded. If the saccade was shorter than 7 visual degrees, was faster than 80 ms or slower than 600 ms, or did not start within 2°of fixation, the trial was discarded. This resulted in an average loss of 19.3% of trials.

**Fig 4:**
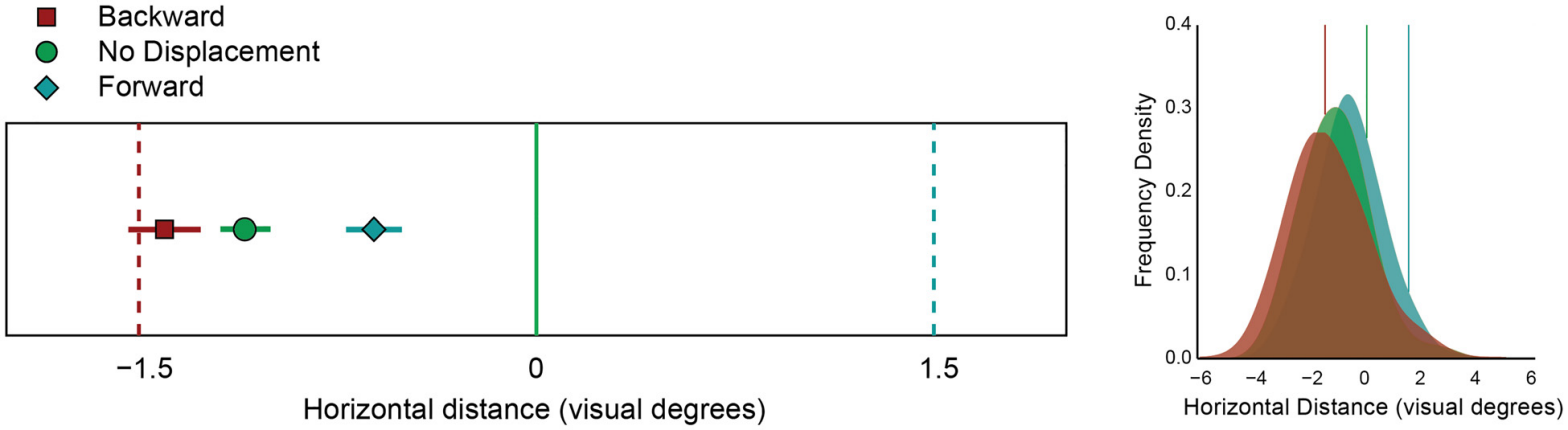
Results of Experiment 3. Left: Mean localization responses in backward, forward and no displacement conditions. Horizontal distance is plotted on the x-axis. The error bars represent 95% within-subject confidence intervals. The solid vertical line indicates the veridical memory location. The dashed vertical lines indicate the displacement size of the saccade target in the backward and forward displacement conditions, respectively. Right: Population density plot of the localization responses relative to veridical location of the memorized stimulus in backward, forward and no displacement. The solid vertical lines indicate the displacement of the saccade target in the backward, no displacement, and forward conditions, respectively.

There was no difference in saccade amplitude between the backward, forward, and no displacement condition in the remaining trials (F(2,14) = 3.66, p =.06). The target disappeared on average 221 ± 6 ms after landing of the first saccade, and localization saccades started on average 313 ms after landing of the first saccade. Mean landing positions of the localization saccade on the horizontal axis relative to the correct cue location for each participant are plotted in Fig 4. There was a general tendency to underestimate the eccentricity of the memory cue location (-1.06 °), and localization was significantly different between Backward, Forward, and No displacement conditions (F(2,14) = 16.92, p<0.001, η_p_^2^ = 0.71), with the average target displacement bias being 21% of total target displacement. Landing points of localization saccades in the vertical direction did not differ among conditions (F(2,14) = 3.10, p =.08). The data were also examined using a Bayes factor ANOVA with default prior scales, comparing the fit of the data under the null hypothesis and the alternative hypothesis. The estimated Bayes factor (alternative/null) suggests that the data are 119 times more likely to occur under the model including the effect of target displacement.

**Fig 5:**
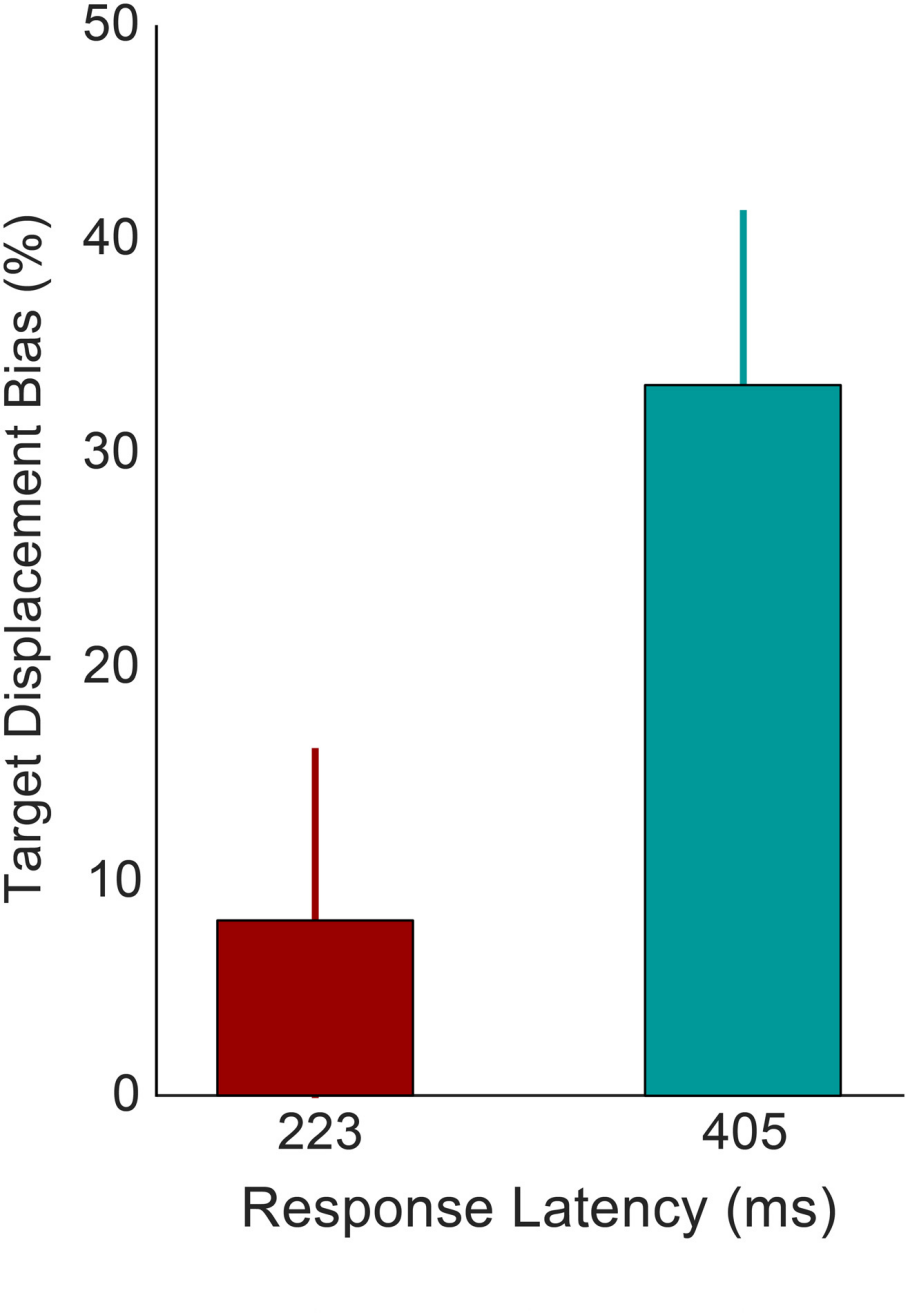
Target displacement bias for fast and slow saccades. The mean effect of target displacement (forward and backward) as a percentage of the total size of displacement, for fast (red) and slow (blue) localization saccades in Experiment 3. Latencies indicate the average interval between landing of the first saccade and start of the localization saccade in the corresponding time bin. The error bars represent 95% confidence intervals.

In Experiment 1 the probe was presented on average 222 ms after the eyes landed on the target. In Experiments 2 it took participants a long time to translate information to a motor program, move the mouse, and click on the location. In Experiment 3 there was a large variability in the timing between the first saccade and localization saccade. Whereas the instruction was to wait with the localization saccade until target offset, part of the saccades were already initiated before or shortly after. This enabled us to compare these very fast localization saccades with slower ones and examine the effect of response latency on localization. For each participant trials were divided into fast and slow responses using a median split based on the time between saccade landing and the start of the saccade to the memorized location. For both bins the target displacement bias (the average effect of target displacement on localization as a percentage of the total size of target displacement) was calculated and plotted in Fig 5. The average latency of the fast responses was 223 ms. The average target displacement bias for these saccades was only 8% of total target displacement. The average latency of the slower responses was 405 ms. For these saccades the average target displacement bias increased to 33% of total target displacement. A within-subjects t-test revealed a significant effect of time bin (t(7) = 6.65, p<0.001, Cohen’s d = 3.02), demonstrating that the effect of target displacement on localization significantly increased over time.

The presence of a corrective saccade did result in the localization saccade to be executed later in time (on average 411 ms after the first saccade, compared to 258 ms for the trials without a corrective saccade). Instead of being a consequence of the increased time available to integrate retinal information, the larger bias observed in trials with slower responses could also be an effect of the larger proportion of corrective saccades in this bin. To check whether the execution of corrective saccade influences localization, we looked at the correlation between the size and direction of the corrective saccade and the localization response. If the target displacement bias is a consequence of the corrective saccade, one would expect the target displacement bias to be larger in trials with larger corrective saccades. However, for none of the participants we found a significant correlation between corrective saccade size and localization responses in either the backward, forward, or no displacement condition.

The contribution of retinal signals in the saccade localization task appears to be similar to that observed in the visual comparison task of Experiment 1. However, localization is largely dependent upon the timing of the response. As more time elapsed between the first and second saccade, the relative contribution of retinal signals increases. Directly after an eye movement the representation seems to be primarily based on the extraretinal signals. The function of this rapid updating might be to allow for fast and accurate actions to be executed, despite the intervening eye movements [42]. However, if there is sufficient time between the first and second eye movement, retinal information seems to be integrated and biases the action plan in the direction of the displaced target.

## Experiment 4

Previous research has demonstrated that transsaccadic displacements of landmark objects can induce an apparent shift of a stationary but briefly blanked target. However, the strength of this illusion depended on the position of the landmark object. In particular, a number of studies have shown that the effect of these landmarks gradually decreases as the distance between the landmark and the target item increases [36,40]. The effect of target displacement on localization demonstrated here might also be dependent on the distance between the saccade target and the memory cue. In fact, we already have some evidence that this effect is not spatially global. In one third of the trials in Experiment 1, 2 and 3 the memorized cue was presented opposite the saccade direction. These trials were not part of the analysis because of the large target-cue distance (between 22.5° and 24.5°) and served only to reduce the predictability of the memory location on saccade target. The analysis of this subset of the data showed an absence of target displacement bias in both Experiment 2 and Experiment 3 (within-participants ANOVAs; both non-significant. In Experiment 1 there were not enough trials to estimate the perceptual null locations), indicating that the contribution of retinal information on memory updating is not spatially global. To investigate the spatial profile of the target displacement bias in more detail we repeated the saccade localization experiment, this time parametrically varying the location of the memory cue across a large area around the saccade target.

### Methods

**Fig 6:**
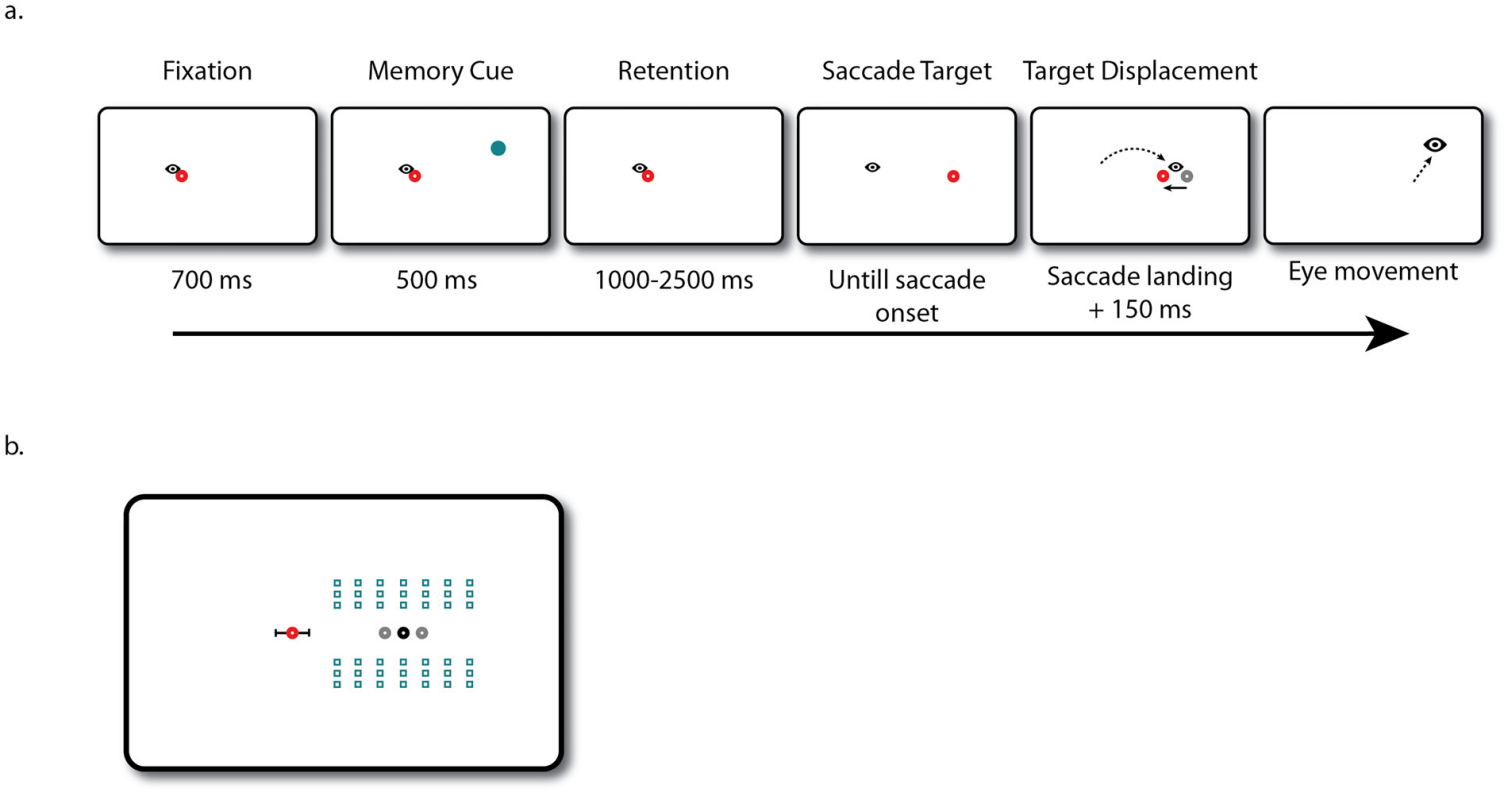
Experimental paradigm of Experiment 4. a. Participants fixated on the red dot and remembered the location of the blue memory cue. After a variable retention interval the dot jumped either to the right or left, signaling that a saccade had to be executed. Once a saccade was detected, the red saccade target could move backward, forward, or remain in place. 150 ms after saccade landing the target was removed, signaling that a localization eye movement had to be executed to the remembered location. b. Overview of the possible memory cue locations (blue) for a rightward saccade. The red fixation dot with error bars indicates the possible fixation locations relative to the saccade target, corresponding to saccades between 9 and 11 visual degrees. The black dot indicates the saccade target location, while the gray dots indicate the location of both a backward and forward displaced saccade target.

Eleven participants aged between 19 and 27 (mean 22, Male: 3) completed on average 968 trials in a saccade localization task similar to Experiments 3. However, instead of picking one out of three possible memory cue locations in the horizontal direction, the memory cue could be presented at one out of seven possible horizontal locations equally spaced between 6° left of the (non-displaced) saccade target and 6° degrees right of it. Once again, vertical distance varied between 2.5° and 4.5° (Fig 6). In contrast to the previous experiments, the saccade target was always located in the same hemifield as where the memory cue was presented. To prevent subjects from predicting the exact location of the saccade target, the distance between fixation and saccade target was randomly varied between 9° and 11°. Whereas in Experiment 3 the target remained on screen for 250 ms after a saccade was detected, in this experiment the target remained visible for 150 ms after the saccade had landed. If a localization saccade was detected before the target offset the trial was discarded and a message signaling that the saccade was too fast appeared on screen.

### Results and Discussion

Trials in which a saccade was detected before the target onset were discarded. If the saccade was shorter than 7 visual degrees, was faster than 80 ms or slower than 600 ms, or did not start within 2° of fixation, the trial was discarded. This resulted in an average loss of 10.1% of trials. There was no difference in saccade amplitude between the backward, forward, and no displacement condition in the remaining trials (F(2,20) = 0.64, p =.54). On average saccades landed 0.42° short of the saccade target. A corrective was made in 17% of the trials in the no displacement condition, 18% of the trials in the backward condition, and in 49% of the trials in the forward condition.

**Fig 7:**
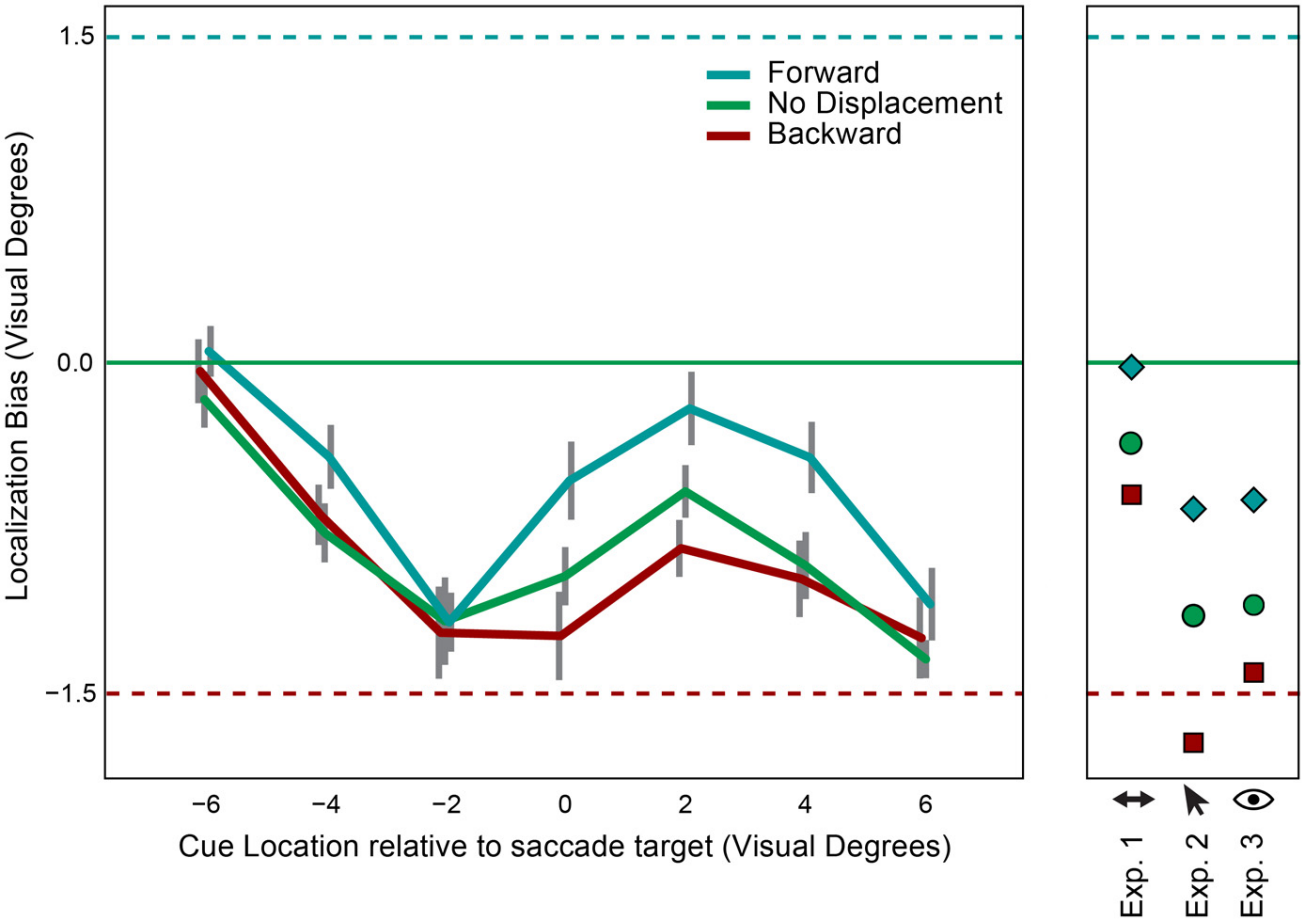
Results of Experiment 4. Left: Mean localization responses in backward (red), forward (blue) and no displacement (green) conditions. Horizontal cue location is plotted on the x-axis. The error bars represent 95% within-subject confidence intervals. The horizontal solid green line indicates the veridical memory location. The horizontal dashed lines on top and at the bottom of the figure indicate the displacement size of the saccade target in the backward (red) and forward (blue) displacement conditions, respectively. Right: For comparison; mean localization responses in backward (red), forward (blue) and no displacement (green) conditions in Experiment 1, 2, and 3. In these experiments the memory cue was presented between 2.5 and 4.5 degrees past the saccade target.

Localization saccades started on average 169 ms after target offset (corresponding to an intersaccadic latency of 319 ms). Mean landing positions of the localization saccade on the horizontal axis are plotted for each cue location in Fig 7. A repeated measures ANOVA with factors target displacement (backward, no displacement, forward) and cue location (-6, -4, -2, 0, 2, 4, 6) revealed a significant main effect of target displacement on localization responses (F(2,20) = 12.33, p<0.001, η_p_^2^ = 0.55). In addition, cue location interacted with target displacement (F(2,20) = 2.87, p<0.01, η_p_^2^ = 0.22), indicating that the effect of target displacement on localization differed across cue locations.

**Fig 8:**
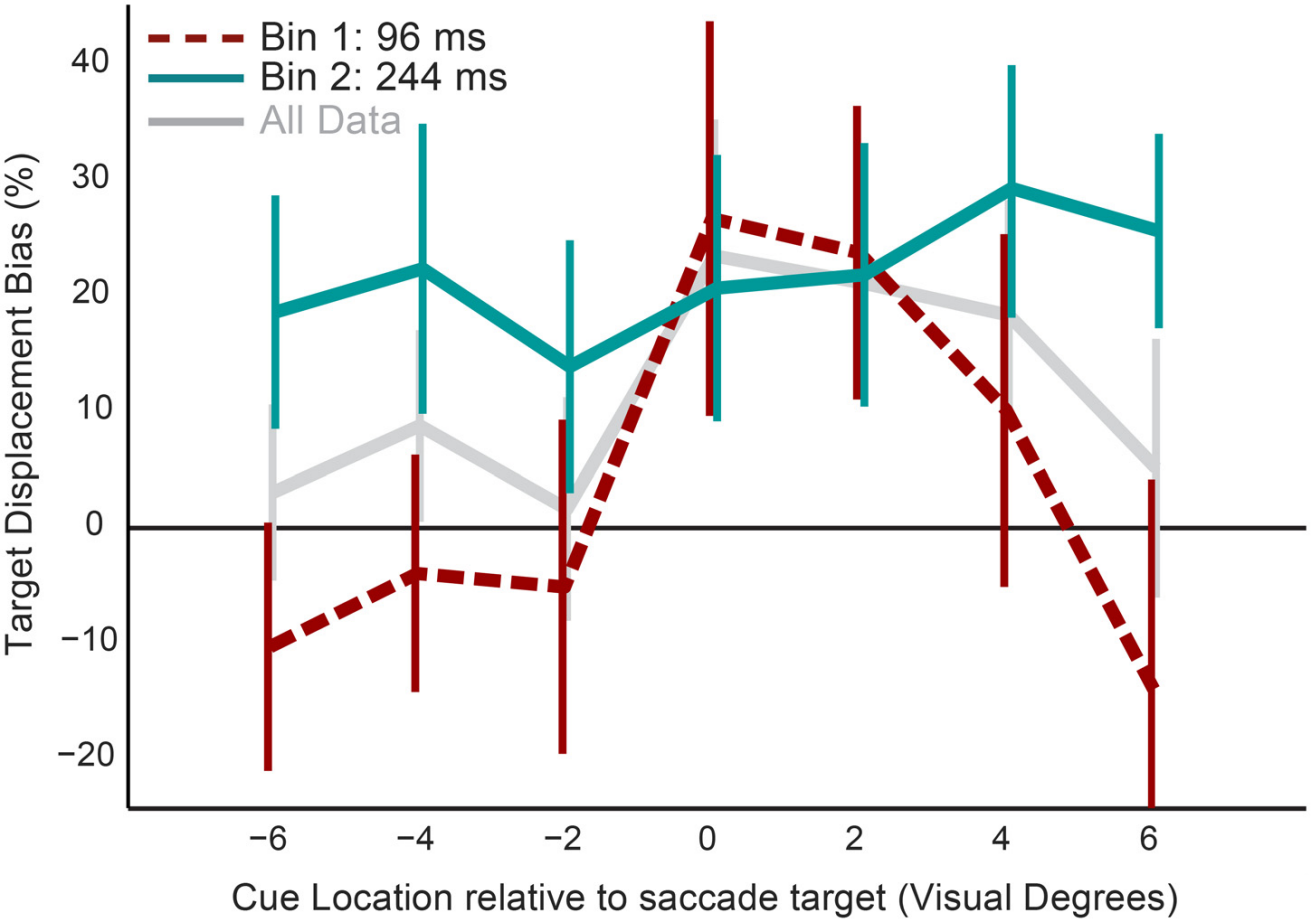
Target displacement bias for fast and slow saccades. The mean effect of target displacement (Forward and Backward) as a percentage of the total size of displacement, at every cue location for fast (red) and slow (blue) localization saccades in Experiment 4. Latencies indicate the average interval between target offset and start of localization saccades in the corresponding time bin. The non-separated data is plotted in light grey. The error bars represent 95% confidence intervals.

For each participant trials were divided into fast and slow responses using a median split based on the time between target offset and the start of the localization saccade. Fast saccades started on average 246 ms after landing of the first saccade, and 96 ms after offset of the saccade target. Localization saccades in the fast bin started on average 394 ms after landing of the first saccade, which corresponded to 244 ms after target offset. For every cue location and time bin the target displacement bias (the average effect of target displacement on localization as a percentage of the total size of target displacement) was calculated and plotted in Fig 8. A within–subjects ANOVA with factors cue location and time bin revealed a significant main effect of time bin on displacement effect (F(1,10) = 20.01, p =.001, η_p_^2^ =.67); there appears to be an increase of target displacement bias as more time passes between target offset and the onset of the localization saccade. In addition, there was a significant interaction between cue location and time bin (within-subjects ANOVA; F(6,60) = 7.52, p<0.001, η_p_^2^ = 0.43); as can be seen from the figure the increase of target displacement bias is larger for cue location further away from the saccade target. Post-hoc analysis reveals that for the fast localization saccades there was only a significant displacement effect for cue locations straight above or below the saccade target or two degrees past the saccade target. For the slower saccades localization is affected by target displacement independent of cue location.

A number of previous studies have demonstrated that the effect of landmark objects on transsaccadic displacement detection gradually decreases as the distance between the landmark and the target item increases [36,40]. The current work focuses on memory retention over saccades and, unlike in previous studies, there was no object present immediately before and after the saccade. Nevertheless, the role of retinal information in trans-saccadic updating is also influenced by the distance between saccade target and memorized item. Whereas displacement detection studies showed a large drop-off of the effect of retinal signals when the horizontal distance between stimuli increased from zero to two degrees, the decrease observed in the current study is much smaller. It should be mentioned that in the previous studies [36,40] both landmark and target were presented on, or very close to, the horizontal meridian. This resulted in the landmark being presented directly above or overlaying the saccade target if the horizontal distance between these stimuli was close to zero, resulting in very large landmark effects for these locations. In the present study saccade target and memorized item were always separated by at least 2.5 degrees of Euclidean distance.

Interestingly, the spatial profile of the target displacement bias changed over time. For the fast localization responses only memorized locations close to the target were biased by the retinal information. However, as the latency of the localization saccades increased, the bias spread to the locations peripheral to the saccade target. The bias produced by the retinal information became spatially global (between 20% and 30% of the total size of target displacement) without affecting the magnitude of the bias for the locations close to the target.

## General Discussion

To maintain an accurate representation of the locations of remembered objects we need to update spatial working memory with every eye movement. In the present study we investigated the role of both extra-retinal and retinal signals in this process. Participants had to maintain a location in memory while making a saccade during which the saccade target could be displaced. Participants had to indicate the memorized location either by a match-to-sample task, a mouse click or by another saccade. Displacement of the saccade target caused a small but systematic bias in the direction of this displacement in all response modalities. This bias was smallest in the saccade task (21% of total target displacement), followed by the match to sample task (24%) and the mouse click task (36%). For the saccade localization task the effect of target displacement increased over time and changed its spatial profile from being initially centered on locations around the saccade target to becoming spatially global. The results show that we neither rely completely on extraretinal nor on retinal information in updating working memory representations across saccades. The relative contribution of retinal signals is not fixed but depends on both the time available to integrate this information as well as the distance between the saccade target and the remembered location.

The small bias introduced by target displacement suggests that retinal information has a limited role in the maintenance of a correct memory representation across saccades. Instead, updating of remembered locations relies to a large extent on the extraretinal signals. Interestingly, studies on normal transsaccadic perception show that these signals are often discarded; as long as objects are continuously present a precise prediction of an object’s post-saccadic retinal location is not crucial as it can easily be relocalized on basis of remembered identity information [39,41,45,46]. For example, when an eye movement accidentally does not land on its target, memorized features of this target are used to drive fast corrective saccades [47]. For the actual detection of object displacements we rely mainly on the position of an object relative to visual landmarks, rather than absolute changes in location [36–40,48]. Objects that are present upon saccade landing will usually serve as a landmark for other objects in the scene. Given that the saccade target was the only landmark in our paradigm, one might expect it to have a large influence on the judged location of the remembered stimulus. However, even when the to-be-memorized stimulus was located in the proximity of the saccade target there was only a minor influence of displacement on the memorized location. Instead, extra-retinal signals appear to be dominant in maintaining a correct representation of memorized stimuli.

This reliance on extra-retinal signals might not be surprising considering the tight link between spatial working memory and the eye movement system. There is a vast body of evidence showing that spatial memory and oculomotor preparation rely on the same neural circuits. Remembered locations interact with saccade preparation [4–7], and experimentally disturbing the preparation of eye movements results in deficits in the maintenance of spatial information [8,9]. Studies in both monkeys and humans demonstrate that saccade goals and remembered locations are represented in the same frontal and parietal brain regions [10–14]. These areas have been characterized as priority maps [22–24]; activity in these maps serves as a pointer to relevant locations. Such locations might be the eye movement goals, but could just as well be an object to attend to or a location to maintain in memory.

If memorized locations are represented in such retinotopically organized priority maps, then how are these maps updated across saccades? The predictive remapping of activity appears to be a suitable mechanism for this. As suggested by Cavanagh and colleagues [21] the main function of remapping might be the predictive updating of attentional pointers in priority maps in order to keep track of relevant locations across saccades. In this way cells might be pre-activated in anticipation of their postsaccadic input. Indeed, remapping properties have mainly been found in areas containing priority maps, such as LIP [10,49], FEF [12,17], and the SC [18,19] in monkeys, and the posterior parietal cortex in humans [20,50]. Moreover, several studies reported the behavioral correlate of such a remapping process; just before an eye movement attention shifts to the retinotopic location a relevant region will occupy after the saccade [26–28]. The updating of remembered locations in the present study might involve the same mechanism.

It has been proposed that prediction of the post-saccadic location of an object is of particularly crucial importance for action control and not so much for perception [42]. The perceptual ignorance displayed in the current study is in line with this; a reasonably large displacement of the saccade target was not perceived by the participants. At the same time the motor system does make accurate corrections for the introduced discrepancy; directly after saccade landing corrective saccades bring the displaced target into the fovea. A similar discrepancy between perception and action was found in a study in which participants simultaneously had to make an eye movement and a pointing movement to a visual target. Participants did not notice perisaccadic displacements of the target, but manual pointing movements were immediately corrected to compensate for the change in location [44]. Although the sensory system seems to be unaware of the discrepancy between the expected and perceived location, the motor system seems to have direct access to this information. Nonetheless, we did not find post-saccadic localization to be more accurate when the tasks involved a mouse or eye movement compared to a perceptual matching task. Localization was most accurate in the eye movement localization task; bias here was only 21% of total target displacement, while the perceptual matching task yielded similar effects (24%). Using a mouse click for localization resulted in the largest bias (36%). The relative contribution of retinal signals to the updating of a remembered stimulus seems to level off around 30%, independent of response modality.

The analysis of saccade latencies in Experiment 3 and 4 did reveal a significant effect of response latency on target displacement bias. The more time elapses between end of the first saccade and the start of the localization saccade, the more interference there is from the post-saccadic retinal input. This result suggests that it takes time to process and integrate retinal information once the eyes have landed. In line with the ideas of Bays and Husain [42], the main function of remapping might be to enable accurate and fast actions without having to process the post-saccadic location of objects with every fixation anew. When the interval between saccade and action is longer there is time to integrate retinal information, which will then also contribute to localization. The findings are in line with previous results demonstrating an optimal integration strategy when having to locate objects across eye movements [51–54]. For example, using a double-step saccade paradigm Munuera and colleagues [51] found an effect of retinal information similar in magnitude to the present study. In addition to the previous findings, we also demonstrate that the time it takes to integrate these different sources of information is dependent on the distance between target and memorized location. The fastest localization saccades were only biased by retinal information if the memorized item was located close to the saccade target. The slower localization saccades were influenced by postsaccadic retinal input independent of the location of the memorized stimulus. It appears that the area affected by retinal signals increases over time. The interaction between the location of the memorized stimulus and the interval during which postsaccadic integration can take place appears to be an important factor in explaining the relative role of retinal and extraretinal signals in memory updating.

Taken together, the current results show that we neither rely completely on extraretinal nor on retinal information in updating working memory representations across saccades. Maintenance of a correct memory representation across saccades seems to rely to a large extent on extraretinal signals. However, the relative contribution of retinal signals is not fixed but depends on both the time available to integrate these signals as well as the distance between the saccade target and the remembered location.

## Supporting Information

S1 File Control experiment. (DOCX)
