# Supplementary material for: The Role of the Oculomotor System in Updating Visual-Spatial Working Memory across Saccades
Source: PLoS One. 2016 Sep 15;11(9):e0161829. doi: 10.1371/journal.pone.0161829 (PMC5025159; doi:10.1371/journal.pone.0161829)
Supplement: S1 File — (DOCX) [file pone.0161829.s001.docx]

**Control experiment**

Although all experiments were conducted in a darkened room, the LCD monitor always emits a dim background illumination. This could have functioned as a stable reference frame, perhaps decreasing the effect of target displacement. To test the viability of this notion, in Experiment 2 we included a control experiment in which we added a bright white reference frame with edges close to the borders of the monitor. Instead of being stable like the real monitor edges this frame was displaced together with the saccade target. This should impair the ability to rely on the monitor’s edges as a spatially stable frame of reference. If the monitor edges indeed served as a landmark diminishing the effect of target displacement we expected to find a larger effect of saccade target displacement when the frame was introduced.

**Methods**

Nine participants aged between 19 and 24 (mean 21, Male: 2) received either money or study credits to participate in a 75 minute experiment, completing 288 trials. The white frame had a size of 20° by 34° and a luminance of 40.3 cd/m^2^ and moved together with the saccade target (Fig A). After completion of the experiment all participants were asked whether they noticed the target displacement on some trials. None of the participants noticed the displacements.


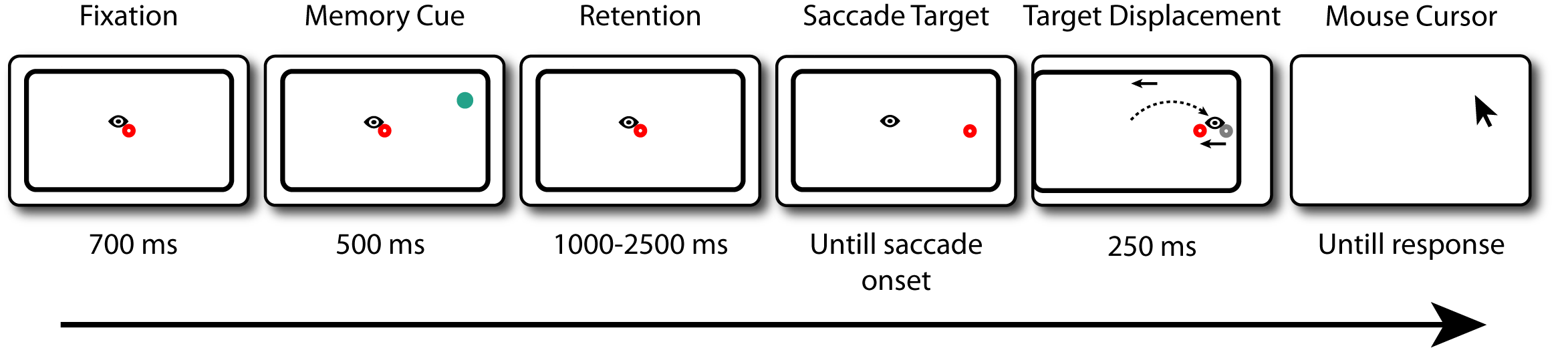


**Fig A.** **Experimental paradigm of the control experiment.** Procedure was exactly the same as in Experiment 2, the only difference being the reference frame that moved along with the saccade target.

**Results and Discussion**

Trials in which a saccade was detected before target onset were discarded. If the saccade was shorter than 7°, was faster than 80 ms or slower than 600 ms, or did start within 2° of fixation were discarded. This resulted in an average loss of 12.2% of all trials. There was no difference in saccade amplitude between the backward, forward, and no displacement condition in the remaining trials (F(2,16) = 2.10, p=.16).

For each participant the mean clicked positions on the horizontal axis relative to the correct cue location are plotted in Fig B. There was a general tendency to overestimate the eccentricity of the cue location by 0.76°. Localization was significantly different among conditions, with the average bias in the backward and forward displacement conditions being 15% of total target displacement (F(2,16) = 8.75, p<.01, η_p_^2^=.52). The estimated Bayes factor (alternative/null) suggests that the data are 13 times more likely to occur under the model including the effect of target displacement. There was no difference in localization in the vertical direction between conditions (F(2,16)= 1.49, p=.26).


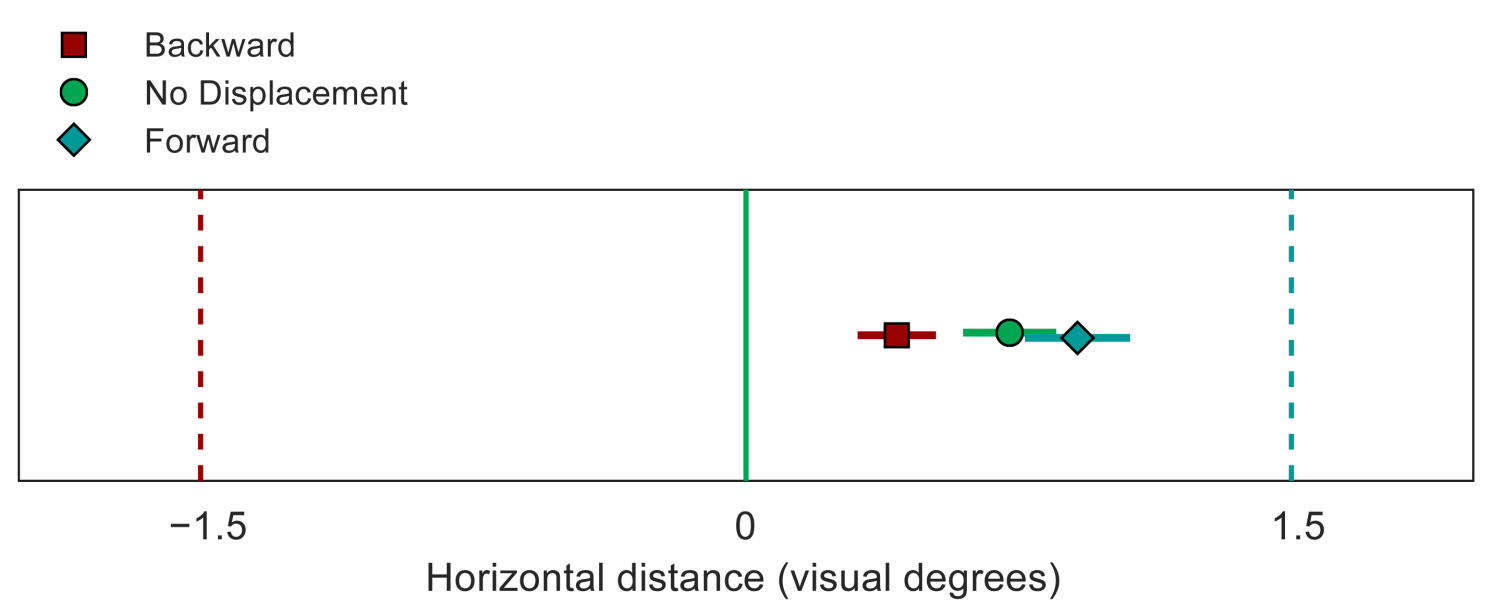


**Fig B. Results of the control experiment.**  Mean localization responses in backward, forward and no displacement conditions in the control experiment (with a reference frame). The horizontal distance is plotted on the x-axis. The error bars represent 95% within-subject confidence intervals. The solid vertical line indicates the veridical memory location. The dashed vertical lines indicate the displacement size of the saccade target in the backward and forward displacement conditions, respectively.

In the control experiment we presented a white frame that moved together with the target. If participants would have used this frame to localize the remembered dot, one would not have expected the average target displacement bias to be much smaller compared to Experiment 2 (36% vs 15% of total target displacement in Experiment 2 and control experiment, respectively). Therefore, the frame did not seem to be used in the post-saccadic calibration of the remembered location, which is in line with earlier findings of Deubel [1]. In his task participants had to indicate the perceived shift of a briefly blanked saccade target, after the perisaccadic displacement of distractor objects. It was demonstrated that displacement of a large reference frame has a considerably smaller effect on the perceived shift of the blanked target than displacement of small distractors in the vicinity of this target. It was proposed that the visual system retains features for only a limited region around the saccade target. Abroad frame, like the monitor edges, may be regarded as an irrelevant background structure, making it a rather ineffective landmark compared to isolated objects.

This, however, does not explain why in the present task the effect of target displacement actually decreased by adding an extra reference object. Possibly, the presence of the reference frame has a more general effect on localization; contrary to the other experiments there is a general overshoot of the localization responses. Responses seem to be ‘pulled’ towards the frame edges. It might be that the reference frame induced a so-called ‘Roelofs effect’; if a target is shown within a frame that is offset to the left or right relative to the participant’s midline, this causes remembered target locations to be shifted in the direction opposite to the offset [2,3]. Since participants’ gaze was located in either the left or the right side of the frame after the saccade, this may have biased location judgments towards the frame edges. This could in turn have led to a ceiling effect: the frame boundaries may have prevented participants from reporting more peripheral localizations.

**References**

1. Deubel H. Localization of targets across saccades: Role of landmark objects. Vis Cogn. 2004;11: 173–202. doi:10.1080/13506280344000284

2. Bridgeman B, Peery S, Anand S. Interaction of cognitive and sensorimotor maps of visual space. Percept Psychophys. 1997;59: 456–469. doi:10.3758/BF03211912

3. Roelofs C. Optische Localisation. Arch Augenheilkd. 1935;109: 395–415.
